# Epidemiological and Socioeconomic Disparities in the 1742–1743 Epidemic: A Comparative Analysis of Urban Centers and Indigenous Populations Along the Royal Road

**DOI:** 10.3390/epidemiologia6020025

**Published:** 2025-05-12

**Authors:** Jorge Hugo Villafañe

**Affiliations:** 1 Departamento de Historia y Filosofía, Universidad de Alcalá, 28801 Alcala de Henares, Spain; mail@villafane.it; 2 Faculty of Medicine, Health and Sport, Universidad Europea de Madrid, 28670 Villaviciosa de Odón, Spain

**Keywords:** plague epidemic, historical demography, colonial South America

## Abstract

Background/Objectives: Epidemics have historically shaped societies, influencing demographic structures, social organization, and economic stability. The 1742–1743 epidemic had a profound impact on populations along the Royal Road (Camino Real), the main colonial corridor between Buenos Aires and Lima. However, its specific demographic and socio-economic effects remain underexplored. This study aims to examine these impacts of the 1742–1743 epidemic through a comparative analysis of urban centers and Indigenous communities. Methods: A historical–comparative approach was employed, analyzing secondary sources including parish records and colonial administrative documents. This study assessed excess mortality and socio-economic consequences across different population groups and settlement types. Results: Mortality rates increased dramatically—up to twelve times the pre-epidemic average in Cordova (Córdoba) and by 45% in Santa Fe—disproportionately affecting Indigenous and enslaved populations. Urban centers experienced severe economic disruption and slow recovery, whereas Indigenous communities and Jesuit missions demonstrated greater resilience. Their communal strategies and early isolation measures contributed to a faster demographic stabilization. Additionally, the epidemic weakened colonial governance in some areas, altering local power structures. Conclusions: The epidemic of 1742–1743 revealed divergent patterns of vulnerability and resilience. Comparative analysis underscores recurring themes in the epidemic response and recovery, drawing relevant parallels with contemporary crises such as COVID-19. Recognizing these historical patterns of adaptation can inform present and future public health strategies. The terminology “plague” is used based on contemporary sources and not confirmed clinically.

## 1. Introduction

The 1742–1743 epidemic marked a pivotal moment in the health and social history of South America, particularly along the Royal Road (Camino Real), the colonial artery connecting Buenos Aires and Lima. While historical documents referred to the outbreak as peste or pestilence, no clinical descriptions or palaeogenetic data exist to confirm its identification as Yersinia pestis by modern standards [1]. However, its demographic and socioeconomic consequences were profound.

Colonial circulation networks such as the Royal Road played a crucial role not only in trade and governance, but also in the spread of infectious diseases. This route connected diverse ecological regions, from coastal lowlands to highland valleys, and functioned as a vector for the transmission of pathogens, goods, and people [2,3]. Originally expanded from pre-Hispanic pathways, its infrastructure supported growing demands for colonial commerce, shaping regional economies and settlement patterns [4,5]. Environmental conditions—including climatic variability and agricultural instability—further intensified the impact of the epidemic, exacerbating pre-existing social inequalities and triggering migration waves and elevated mortality [6]. The effects were particularly acute in Indigenous communities and Jesuit missions, whose spatial concentration and limited mobility created ideal conditions for disease transmission [7,8].

Recent studies reveal dramatic demographic consequences along this corridor. In Cordova, mortality rose twelvefold in May 1743, while Santa Fe saw a 45% increase by December of the same year [9]. The epidemic disproportionately affected Indigenous and enslaved populations, intensifying social vulnerability. Although urban centers imposed containment measures aligned with colonial governance, Jesuit missions and Indigenous communities developed alternative strategies rooted in communal organization and spiritual frameworks.

The outbreak’s diffusion mirrored patterns of trade and mobility, underscoring the limitations of public health measures in a colonial setting. Beyond its epidemiological impact, the epidemic catalyzed significant transformations in labor systems, governance, and social hierarchies, offering insight into broader patterns of resilience and institutional fragility.

This article explores the differential impact of the 1742–1743 epidemic along the Royal Road through a comparative analysis of urban centers and Indigenous communities. It examines how demographic and socioeconomic disparities shaped vulnerability, mortality, and recovery. Drawing from historical demography, epidemiology, and medical anthropology, this study assesses excess mortality and interprets the crisis through both archival and historiographical lenses. The findings contribute to a more nuanced understanding of epidemic dynamics in colonial Latin America and offer reflections relevant to present-day public health challenges.

## 2. Materials and Methods

### 2.1. Study Design

This study employs a historical–comparative approach based on the analysis of published data in the academic literature to evaluate the impact of the 1742–1743 plague epidemic on different settlements along the Royal Road. The research compares findings reported in previous studies on urban populations with those related to Indigenous communities and Jesuit missions, aiming to identify differences in demographic evolution and institutional and community responses to the crisis.

Given that the available information is dispersed across various studies, a strategy of secondary data synthesis and contrast has been implemented, assessing the methodological quality of the analyzed works and their internal consistency. A combination of historical demography techniques and qualitative analysis is employed to interpret the patterns reported in the literature and situate them within a comparative framework.

### 2.2. Data Sources

This research aggregates and synthesizes data from a diverse array of secondary sources, including published demographic reconstructions based on parish records and colonial administrative documentation. These sources were carefully selected for their historiographical significance and methodological rigor. This study focuses on cities for which consistent and longitudinal mortality records are available—Cordova and Santa Fe—and incorporates qualitative information on Jesuit missions in the Chaco region (e.g., Abipones and Mocovíes), which are referenced to highlight contrasting epidemic experiences in Indigenous and mission settings.

Given the inherent methodological constraints and potential biases in the data collection and presentation, an exhaustive critique of their reliability has been undertaken. This critical evaluation encompasses considerations such as the representativeness of the samples, methodologies employed for demographic reconstruction, and the interpretative frameworks utilized. This ensures that the conclusions drawn reflect the complexities of the historical context.

#### 2.2.1. The Main Categories of Reviewed Sources Include

- Demographic studies and previous statistical analyses: published works estimating mortality rates, the population decline, and demographic recovery, typically based on parish records and administrative reports.
- Historical analyses of the epidemic and its repercussions: research examining the social and economic dimensions of the epidemic, including governmental and community responses.
- Historiographical sources on Indigenous populations and Jesuit missions: studies focusing on the epidemic’s impact in Indigenous and mission settings, enabling comparisons with urban cases.
- The comparative literature on epidemic crises in Latin America: publications providing contextual and regional insights into epidemic patterns during the colonial period.

#### 2.2.2. Contextualization of Disease Dynamics

Given the absence of standardized diagnostic records in colonial South America, terms such as “peste” were used historically, but should not be equated automatically with plague as currently defined.

Excess mortality was estimated by comparing monthly deaths during the epidemic years (1742–1743) with the pre-epidemic averages (1740–1741). The lack of data on contagion rates prevents the calculation of case fatality rates (CFR).

### 2.3. Analytical Strategy

#### 2.3.1. Quantitative Synthesis and Comparison

- Mortality estimates and recovery trajectories are compared across the available studies, with particular focus on Cordova and Santa Fe, the only urban centers for which monthly mortality data during the pre-epidemic (1740–1741), epidemic (1742–1743), and post-epidemic (1744–1745) phases are available.
- Differential demographic patterns are identified between urban centers and Indigenous/mission communities, with attention to post-crisis population stabilization.
- Previous studies’ adjustment models for under-reporting and data incompleteness are considered to evaluate their relevance across varied demographic settings.
- Excess mortality was estimated by comparing monthly deaths during the epidemic years (1742–1743) with the average monthly deaths from 1740–1741, using parish record data. While this method does not account for population growth or migratory movements, it provides a comparative baseline to assess the demographic impact of the epidemic across the regions and settlement type.

#### 2.3.2. Qualitative Analysis and Historiographical Interpretation

- Historiographical interpretations are critically reviewed, balancing structural explanations (e.g., trade routes, colonial policies) with perspectives that emphasize agency in Indigenous and community responses.
- Institutional responses (e.g., quarantine policies, ecclesiastical measures) are contrasted with those developed by Indigenous communities and Jesuit missions.
- Findings are contextualized within broader patterns observed in other Latin American epidemics to identify both shared characteristics and local specificities.

## 3. Results

### 3.1. Excess Mortality and Demographic Distribution

The analysis confirms a substantial increase in mortality during the 1742–1743 epidemic across both urban centers and Indigenous communities. In Córdoba, mortality in May 1743 rose to more than twelve times the pre-epidemic monthly average, while in Santa Fe, a peak was recorded in December of the same year. Although mortality rates were relatively lower among Indigenous groups, the epidemic deeply disrupted their social and economic structures, intensifying labor coercion and altering demographic balances [10]. To improve transparency, a summary of the findings is provided in Table 1.

**Figure 1:**
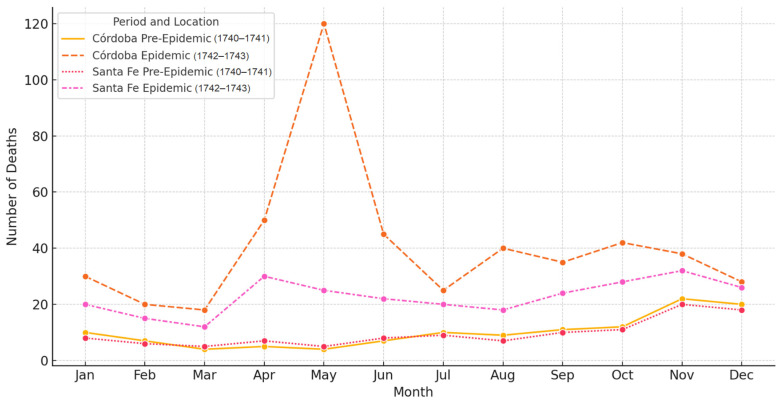
Monthly mortality in Córdoba and Santa Fe during the 1742–1743 epidemic, compared to pre-epidemic levels (1740–1741).

Monthly mortality trends are visualized in Figure 1, which highlights the sharp seasonal mortality peak in Córdoba during the epidemic. While the figure does not illustrate spatial dissemination, it reflects the intensity and timing of the outbreak in urban centers along the Royal Road.

Urban centers were particularly vulnerable due to the high population density and intense mobility. In contrast, Jesuit missions, such as those of the Abipones and Mocovíes, reported mortality increases of approximately 150%, despite initial isolation efforts. These figures suggest that although containment strategies may have delayed transmission, they were ultimately insufficient to prevent the demographic impact [11].

Population loss in Indigenous communities disrupted kinship networks, labor systems, and local governance, contributing to long-term socioeconomic instability. Environmental factors—including climatic fluctuations and possible rodent population surges—likely exacerbated transmission dynamics, consistent with patterns documented in other endemic regions of the Americas [12,13].

### 3.2. Socioeconomic Impacts and Community Responses

Institutional responses to the epidemic differed significantly between colonial urban centers and Indigenous settlements. In cities like Córdoba and Santa Fe, municipal and ecclesiastical authorities implemented quarantine measures, travel restrictions, and religious interventions. However, the persistent flow of goods and people along the Royal Road often undermined these efforts [2]. The epidemic also triggered structural changes in the labor system. Labor shortages, particularly in urban areas, led to increased reliance on forced Indigenous and African labor. This reorganization reinforced existing inequalities, disproportionately affecting marginalized populations [14].

In contrast, Indigenous communities and Jesuit missions implemented more cohesive and localized strategies. Rooted in self-governance and religious communalism, their approach emphasized social cohesion, resource control, and strict limits on external contact [15,16]. These features enabled more effective isolation and the supported internal redistribution of food and labor, which contributed to a relatively faster recovery than that observed in colonial urban economies [12,13].

Spanish control weakened in certain regions during the crisis, facilitating the rise of informal economies and trade networks operating beyond colonial regulation. Medical disparities also intensified social inequality: while urban elites had sporadic access to European medical practices, Indigenous and enslaved populations relied on traditional healing systems, reinforcing epistemological and material asymmetries [17].

### 3.3. Comparison of Resilience and Recovery

Recovery trajectories diverged sharply after the epidemic. Urban centers experienced slow and uneven demographic and economic recovery, constrained by population loss and institutional rigidity. Indigenous communities, in contrast, benefited from communal land management and inter-mission support networks, which fostered more effective adaptation [13].

**Figure 2:**
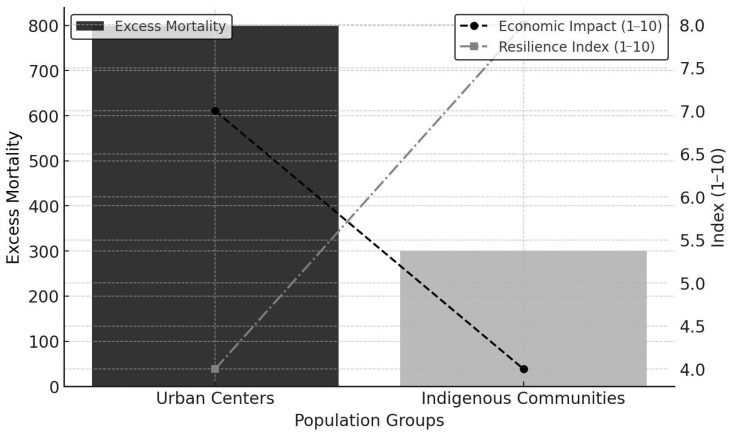
Comparative impact of the 1742–1743 epidemic on urban centers and Indigenous populations. The figure illustrates disparities in excess mortality (bars), economic disruption (dashed lines), and resilience. Urban centers experienced higher mortality and greater disruption, whereas Indigenous communities showed lower mortality and relatively greater recovery capacity.

Not all missions recovered equally; some faced an irreversible demographic decline, resulting in administrative restructuring or eventual closure [11]. The epidemic also magnified tensions around communal organization. While Jesuit missions demonstrated adaptability, their model later attracted political criticism, contributing to the suppression of the Jesuit order (Figure 2).

Finally, comparisons with modern pandemics, such as COVID-19, reveal enduring patterns: the disproportionate suffering of vulnerable populations, fragmented institutional responses, and profound economic reconfigurations [18,19]. Environmental determinants—such as climate variability and ecological disruption—also emerge as persistent drivers of epidemic dynamics across both historical and contemporary settings [13].

## 4. Discussion

The 1742–1743 plague epidemic along the Royal Road between Buenos Aires and Lima was a pivotal event in the demographic and socio-economic history of colonial South America. Through the analysis of parish records and secondary historical sources, this study has identified significant mortality patterns, social reconfigurations, and long-term demographic consequences. The findings underscore the crucial role of trade networks and mobility in disease dissemination, as well as the disparities in vulnerability and resilience between urban centers and Indigenous communities.

### 4.1. Disparities in Mortality and Social Consequences

The epidemiological characteristics of the 1742–1743 outbreak are consistent with a predominance of bubonic plague, likely propagated through rodent–flea cycles and facilitated by commercial mobility along the Royal Road. While no definitive clinical documentation exists, the observed monthly mortality patterns—marked by a significant increase beginning in autumn (April–May), a peak during the winter months (June–August), and persistence into spring and early summer—reveal a clear seasonal component. This pattern, in conjunction with the widespread use of the historical term peste, and the rapid geographic spread via trade routes, reinforces the hypothesis of the plague etiology. Previous studies have established that plague transmission in colonial South America was often linked to climatic variability that affected rodent ecology, particularly in contexts of increased rainfall and intensified agricultural activity. The prevalence of rats and fleas in densely populated urban areas and Jesuit missions provides further support for this interpretation. Although alternative diagnoses—such as epidemic typhus or typhoid fever—remain plausible, the intensity, extent, and temporal profile of mortality align more closely with historical plague dynamics in the early modern Americas.

This epidemiological framework informs the analysis of demographic and social disparities observed across settlement types. One of the central findings of this study is the marked contrast in mortality rates between urban centers, Indigenous settlements, and frontier populations. In cities such as Cordova and Santa Fe, mortality rates surged—reaching up to twelve times the pre-epidemic baseline—with Santa Fe recording its peak in December 1743 [10]. Marginalized groups, particularly Indigenous peoples and enslaved individuals, were disproportionately affected, revealing how pre-existing social hierarchies and inequalities shaped exposure and vulnerability during the crisis [10].

The epidemic also catalyzed broader transformations in settlement organization and social stratification. While urban elites focused on restoring economic order and institutional control, Indigenous and rural communities developed adaptive responses centered on isolation and communal resilience [11]. Jesuit missions, which exhibited comparatively lower mortality, leveraged their centralized organizational structures to implement more effective containment measures [13]. Nonetheless, some Indigenous settlements experienced enduring demographic disruptions, including population decline, labor shortages, and displacement, which undermined long-term community stability.

In frontier regions such as the Chaco and the Southern Frontier—historically characterized by commercial interactions, conflict, and negotiation—the epidemic likely altered power relations between colonial authorities and Indigenous groups. Although archival data remain fragmentary, existing research suggests that the epidemic-induced population decline contributed to shifts in Indigenous resistance strategies and Spanish military policies [20,21]. The demographic impact of the epidemic may have accelerated socio-political reconfigurations, weakening Indigenous groups previously engaged in resisting colonial expansion and intensifying disputes over land, labor, and resource control.

### 4.2. The Role of the Royal Road in Epidemic Transmission and Regional Dynamics

The Royal Road served as a principal conduit not only for economic flows, but also for the dissemination of epidemic diseases, although specific clinical identification remains speculative. The continuous circulation of goods, people, and livestock between Buenos Aires and Lima contributed to the dissemination of pathogens along this trade artery, highlighting the interplay between economic infrastructure and epidemiological risk [8].

Beyond its economic role, the Royal Road was a central mechanism of colonial governance, structuring territorial organization and consolidating Spanish authority over vast regions. It enabled communication and administrative coordination between urban centers, Indigenous settlements, and frontier territories. However, the maintenance and expansion of this infrastructure faced persistent challenges, including Indigenous resistance, environmental obstacles, and logistical difficulties in sustaining long-distance transport networks [3]. The 1742–1743 epidemic exposed the fragility of these colonial communication systems, as quarantines and travel restrictions proved largely ineffective in halting disease transmission across this highly interconnected landscape. The inability to control mobility during the outbreak underscores the limitations of colonial governance in enforcing public health measures amid widespread economic and political dependencies on trade routes.

The Cartas Anuas offer critical qualitative evidence regarding the interplay between epidemic outbreaks and patterns of mobility along the Royal Road. Contemporary testimonies describe widespread population displacements, as Indigenous groups abandoned missions and settlements in attempts to evade infection—a phenomenon similarly reflected in the demographic disruptions recorded in Santa Fe [16]. These migratory movements not only accelerated the spread of disease, but also contributed to the destabilization of regional economies and colonial administrative systems. In addition, the Jesuit interpretation of the epidemic as a manifestation of divine punishment shaped communal responses, reinforcing religious frameworks for understanding the crisis and motivating extensive spiritual mobilizations [15]. The incorporation of these accounts complements quantitative mortality data with socio-cultural insights, allowing for a more comprehensive understanding of the epidemic’s broader human impact.

In frontier regions such as the Chaco and the Southern Frontier, where Spanish control was more tenuous, the epidemic likely exacerbated tensions over land, trade, and Indigenous labor systems. The expansion of the Royal Road had already disrupted traditional Indigenous economies, and the epidemic further altered population densities and labor availability in these contested regions [22,23]. The colonial response in these zones was further complicated by frontier instability, as declining Indigenous populations due to disease created power vacuums that reshaped alliances, conflicts, and economic adaptations.

The differential impact of the epidemic on various populations was closely tied to their position within the Royal Road’s economic structure. Urban centers, deeply integrated into colonial markets, suffered severe economic downturns due to labor shortages and trade disruptions [7]. The decline in the workforce significantly affected agricultural production, artisanal industries, and commerce, leading to inflation in essential goods and heightened social tensions. In contrast, Indigenous settlements and Jesuit missions, though affected by the epidemic, demonstrated greater resilience due to their communal economies and localized resource management systems. Their relative economic autonomy from colonial trade structures allowed for more effective subsistence strategies, reinforcing self-sufficiency and internal cohesion. However, this resilience was not absolute, as certain Indigenous communities experienced long-term economic destabilization due to labor shortages and demographic imbalances [3].

### 4.3. Institutional Responses and Their Limitations

The colonial administration’s response to the epidemic reflects the limitations of the public health infrastructure in the eighteenth-century Spanish Empire. Authorities implemented measures such as quarantines, road closures, and religious ceremonies in an attempt to mitigate the spread of the disease [2]. However, these efforts were largely ineffective, as commercial networks and mobility along the Royal Road facilitated continuous transmission. This underscores the inherent challenges of epidemic containment in a colonial context, where trade and governance were deeply intertwined with mobility [4].

The epidemic also highlighted the importance of community-based responses in crisis management. While urban religious institutions coordinated public prayers and processions, Indigenous communities relied on healing rituals and traditional knowledge to combat the disease [17]. European and Indigenous responses to health crises reflected different epistemologies; this duality complicated public health measures.

### 4.4. Economic Disruptions and Post-Epidemic Transformations

The epidemic had a profound impact on colonial economic structures, disrupting the labor supply, trade networks, and production systems. In urban centers, workforce shortages led to a crisis in agricultural and artisanal production, exacerbating food scarcity and driving up the prices of essential goods [14]. To compensate for demographic losses, there was an increased reliance on coerced Indigenous labor and enslaved workers, reinforcing patterns of exploitation that persisted in the following decades.

Conversely, Jesuit missions exhibited greater economic stability due to their communal production model. However, while some missions managed to sustain their economic activity, others were unable to withstand the demographic impact of the epidemic, leading to their restructuring or dissolution. These differences in recovery processes reflect how social and economic organization influenced the adaptive capacity [11]. As observed in contemporary epidemics, communities with localized production structures and internal redistribution mechanisms displayed greater resilience to economic disruption.

### 4.5. Comparative Perspective: Lessons from Historical and Contemporary Epidemics

Patterns of vulnerability, institutional fragility, and socio-economic restructuring during the 1742–1743 epidemic show parallels with responses to contemporary pandemics such as COVID-19. In both historical and contemporary contexts, the most vulnerable populations suffered the most severe epidemic consequences, institutional responses were often fragmented, and socioeconomic structures underwent significant post-crisis transformations [19].

Moreover, environmental factors have consistently shaped epidemic dynamics. As evidenced by historical records, climate variability and ecological changes played a role in the transmission of the 1742–1743 epidemic, particularly through shifts in rodent populations and agricultural expansion [12,13]. Similarly, modern research has demonstrated how climate change continues to influence the spread of infectious diseases today. These parallels underscore the relevance of historical models in understanding and preparing for future health crises.

### 4.6. Implications for Historiography and Public Health

The findings of this study contribute to the historiography of epidemics in colonial Latin America by offering a comparative perspective on epidemiological and socioeconomic disparities. By integrating historical demography, economic history, and medical anthropology, this research provides a nuanced understanding of how different populations along the Royal Road experienced and responded to the epidemic.

Additionally, this study highlights the importance of interdisciplinary approaches in epidemic research. The combination of a demographic analysis, historical epidemiology, and socio-economic history allows for a more comprehensive assessment of past health crises and their long-term consequences. Future studies should continue exploring the intersection between disease, colonialism, and social inequality to better understand the complex legacies of historical epidemics.

## 5. Conclusions

The 1742–1743 plague epidemic along the Royal Road between Buenos Aires and Lima was a pivotal event in the demographic and socioeconomic history of colonial South America. This study highlights the epidemic’s disproportionate impact on various population groups, exacerbating pre-existing inequalities and reconfiguring economic and social structures. Through a comparative analysis of urban centers and Indigenous communities, key disparities in mortality, response strategies, and post-epidemic recovery have been identified.

The findings reveal a marked contrast in mortality rates between urban settlements and Indigenous communities. Cities such as Cordova and Santa Fe suffered severe demographic losses due to a high population density and mobility, while Indigenous groups and Jesuit missions implemented containment strategies that mitigated the impact. Nonetheless, the epidemic had long-term demographic consequences, affecting labor availability, migration patterns, and economic sustainability. Institutional responses, including quarantines and religious interventions, proved largely ineffective, while localized community strategies demonstrated greater resilience.

Economically, the crisis disrupted trade and labor markets, particularly in urban centers where workforce shortages led to production declines and inflation. Meanwhile, Indigenous settlements and Jesuit missions, operating under communal economic structures, exhibited greater stability, though some experienced long-term decline. Additionally, the epidemic weakened colonial administrative control in certain regions, fostering informal economies and alternative trade networks, illustrating how health crises can drive broader socioeconomic transformations.

A comparative perspective with contemporary pandemics, such as COVID-19, underscores recurring patterns in the epidemic impact and societal response, particularly regarding the heightened vulnerability of marginalized populations and the fragmented nature of institutional responses. These parallels highlight the necessity of integrating historical insights into modern public health and policy frameworks.

By integrating historical demography with economic history and medical anthropology, this study not only enriches the historiography of colonial Latin America, but also offers valuable lessons for understanding the dynamics of epidemics in historically interconnected regions. Future research should further examine regional variations in epidemic responses and explore how governance, Indigenous knowledge systems, and economic structures influenced resilience and recovery along the Royal Road.

## Figures and Tables

**Table 1:** Comparative mortality rates during the pre-epidemic, epidemic, and post-epidemic phases of the 1742–1743 plague in the city of Cordova and Santa Fe.

Month	Pre-Epidemic, 1740–1741	Epidemic, 1742–1743	Post-Epidemic, 1744–1745
January	17 (7.0%)	54 (9.7%)	21 (7.8%)
February	16 (6.6%)	28 (5.1%)	19 (7.1%)
March	11 (4.5%)	32 (5.8%)	20 (7.4%)
April	24 (9.9%)	59 (10.6%)	22 (8.2%)
May	10 (4.1%)	52 (9.4%)	22 (8.2%)
June	17 (7.0%)	46 (8.3%)	25 (9.3%)
July	19 (7.8%)	37 (6.7%)	26 (9.7%)
August	21 (8.6%)	49 (8.8%)	24 (8.9%)
September	15 (6.2%)	47 (8.5%)	23 (8.6%)
October	19 (7.8%)	50 (9.0%)	25 (9.3%)
November	34 (14.0%)	49 (8.8%)	15 (5.6%)
December	40 (16.5%)	51 (9.2%)	27 (10.0%)
Total	243 (100%)	554 (100%)	269 (100%)

## Data Availability

MDPI Research Data Policies.
